# What Is *Chalky*? Investigating Consumer Language and Perception of Fine Particles in Beverages Containing Pea and Potato Starch

**DOI:** 10.3390/foods13121852

**Published:** 2024-06-13

**Authors:** Kai Kai Ma, Gregory R. Ziegler, Helene Hopfer, John E. Hayes

**Affiliations:** 1 Sensory Evaluation Center, College of Agricultural Sciences, The Pennsylvania State University, University Park, PA 16802, USA; kkm5269@psu.edu (K.K.M.); hxh83@psu.edu (H.H.); 2 Department of Food Science, College of Agricultural Sciences, The Pennsylvania State University, University Park, PA 16802, USA; grz1@psu.edu

**Keywords:** chalky, texture, mouthfeel, salivary flow

## Abstract

Despite its importance as an undesirable food texture, the phenomenon of chalkiness remains understudied. *Chalky* sensations presumably arise from fine particulates found in foods, but semantic overlap with other common descriptors of small particles, like gritty or sandy, is unclear. Here, we compare the usage of *Chalky* with related descriptors, and determine the effect of particle size, concentration, and xanthan content on *Chalky* ratings in a model beverage. A 2^3^ factorial design with starch particle size (D_90_ = 33.8 and 64.6 µm), starch concentrations (10 and 20% *w*/*v*), and xanthan content (0.075 and 0.15% *w*/*v*) was used. Participants’ salivary flow rate was also assessed. A multi-sip taste test was performed where naïve consumers (n = 82; 39% men, 60% women; age range = 18–79 years) rated the intensity of *Chalky*, *Powdery*, *Gritty*, *Sandy*, *Mouthdrying*, and *Residual mouthcoating* at 0, 30, and 60 s after each of three consecutive sips. All attribute ratings were highly correlated, with *Chalky*, *Powdery*, and *Residual Mouthcoating* being more closely correlated with each other than *Gritty* or *Sandy*. Although *Chalky* was still reported 60 s after consumption, no evidence of build-up was found with repeated sips. A larger size and higher concentration increased *Chalky* ratings, with the low-salivary-flow group reporting greater ratings for *Chalky* relative to the high-flow group. Our results suggest consumer percepts of small particles are overlapping but not entirely redundant. This suggests researchers and product developers should carefully distinguish between these descriptors when trying to understand consumer perception of food products containing fine particles.

## 1. Introduction

Chalky is a sensory attribute arising from small particles in food that is typically seen as a defect. It is as important to study unfavorable sensations as desirable ones, as minimizing such defects can increase the acceptability of food [1]. However, chalkiness is understudied, both in terms of how consumers use this term as well as the mechanisms responsible. Commonly consumed foods that have been described as *chalky* include yogurt drinks, plant-based milk, fiber-fortified beverages, and medical nutrition products [2,3,4,5,6,7]. While many of these fortified foods provide extra nutrients that help meet consumers’ needs, chalkiness lowers the palatability of these products. For example, Brückner-Gühmann et al. [8] found that *chalky* and *floury* attributes from the addition of oat protein concentrate to oat-based yogurt decreased liking. Multiple studies have defined *chalky* as a dry, fine, powdery particulate sensation [4,9,10,11]. Other terms have also been used to describe the perception of fine particles in foods including *Gritty*, *Powdery*, and *Sandy*. Many of these terms have been defined similarly, as sensations associated with small particles, with references to flours [12,13,14,15]. Some of these also include descriptions related to approximate particle sizes, including sand-like or flour-like. However, it is unclear how consumers distinguish among these terms.

Stimuli that elicit chalky sensations are not well understood. In prior work, it has been regarded as a mouthfeel [2] or texture attribute [16]. A recent study by van der Stelt et al. [17] investigated the mouthfeel of medical nutrition products; they suggested other sensations like mouthdrying, residual mouthcoating, lip-coating, and throat-coating are also related to the perception of fine particles. *Chalky* and *Powdery* were considered not only as textures in the mouth pre-swallowing but also as mouth sensations after swallowing. It is unclear whether *Chalky* is related to mouthcoating, lip-coating, or throat-coating, in terms of how consumers use these terms or in terms of the mechanisms underlying these sensations. Many studies that attempt to quantify *Chalky* sensations only obtain a single rating immediately after sampling (e.g., Allgeyer et al. [2], Chakraborty et al. [4], Drake et al. [5], Shewan et al. [10], van der Stelt et al. [17]). However, these sensations may build over time. For example, Withers and colleagues [18] used sequential profiling to understand the temporal effect of repeated consumption on *Chalky* mouthfeel of whey and casein protein-fortified dairy beverages. With repeated consumption, they found there was a buildup in chalky ratings over time. This suggests it may be important to test how chalky sensations may change over time with repeated consumption in other food products, especially if one assumes that the particles causing this sensation might accumulate in the mouth over time.

Particle size, shape, and concentration have been implicated as key factors in the perception of fine particles in food. Early work by Tyle [19] highlighted the importance of particle size, shape, and hardness on the perception of grittiness in a suspension model. By having participants taste suspensions of inedible particles (i.e., garnet, micronized polyethylene, and mica coated with titanium dioxide) with a mean size of 5 to 80 μm, they found that increasing particle size increased grittiness, but only for harder particles. Soft and round particles were not perceived as gritty up to 80 μm, while hard and angular particles from 11 to 22 μm were perceived as gritty. Using similar methods with particles of microcrystalline cellulose (6–79 μm), Imai [20] found that particle concentration was the most important factor determining the perception of grittiness, followed by dispersion medium and particle size. The perception of grittiness increased with a higher particle concentration and lower dispersion medium viscosity. Subsequently, Imai et al. [21] showed a similar result of the effect of particle size on the perception of the graininess (similar to grittiness) of food particles. This suggests the particle size as well as the dispersion viscosity each influence the perception of fine particles, at least for gritty and grainy sensations; here, we reasoned that they may also impact the perception of *Chalky*.

Other work suggests terms like *Mouthcoating* and *Mouthdrying* have been used to describe chalky foods including fiber- and whey protein-enhanced foods [4,13,18]. Shewan et al. [10] found that a greater hardness of microgel particles increased the perception of particle size and chalkiness and decreased smoothness. An increase in particle concentration elevated the perception of smoothness, thickness, clearance, cloying, and mouthcoating. In a more complex model beverage fortified with soluble and insoluble fiber, a higher soluble fiber concentration did not increase the perception of chalkiness, whereas insoluble fiber did [4]. Samples with more insoluble fiber increased the perception of particles, mouthcoating, lingering, dryness, and chalkiness, where dryness had a higher score than chalkiness when a small amount of dietary fiber was added. Consistent with prior work, a larger particle size increased the perception of particles in the mouth [19,20,21]. The addition of casein and milk protein in dairy beverages also increased chalky, mouthcoating and mouthdrying perceptions [18]. If sensations of mouthcoating, mouthdrying, and chalkiness are caused by the retention of fine particles in the mouth after swallowing, then salivary flow rate, by aiding the removal of particles from the mouth after swallowing, may influence customers’ perception, possibly with differential effects across individuals. Prior work on oral tribology shows that saliva acts as a lubricant, reducing the friction caused by the presence of food particles [22]. The degree of lubrication can determine the readiness of the food for swallowing [23]. Saliva flow and composition are known to affect orosensory perception [24,25,26,27,28,29]. For example, the addition of saliva or amylase solution in custard affected mouthfeel attributes including melting, thickness, and creamy [24]. Guinard et al. [23] found that increased saliva flow can enhance the perception of the adhesiveness of peanut butter and the cohesiveness of cracker mass. Accordingly, we reasoned that salivary flow rate may also impact the perception of chalkiness.

The present study attempts to understand potential mechanisms of chalky sensation, using a model beverage sipped multiple times. The effect of starch granule size, concentration, and xanthan content were evaluated. Finally, we also evaluated the role of salivary flow rate on chalky sensations. Specifically, we hypothesized that (1) *Chalky* and *Powdery* are similar sensations while *Gritty* and *Sandy* are a different set of related but distinct sensations; (2) *Chalky* ratings would build over time with consecutive consumption; (3) participants with a high salivary flow rate would give lower ratings for *Chalky* relative to low-flow individuals.

## 2. Materials and Methods

### 2.1. Ethics Statement

All procedures, including screening, recruitment, consent, and compensation, were approved by the Institutional Review Board (IRB) at The Pennsylvania State University as an expedited protocol (STUDY00022256).

### 2.2. Participant Recruitment and Consent

A total of 99 participants were recruited from the campus and community surrounding the Pennsylvania State University in State College PA. Sensory tests were carried out in individual booths in the Sensory Evaluation Center in the Erickson Food Science Building at Penn State. All participants provided informed consent and were paid a small cash incentive for their time. Exclusion criteria included being below 18 years of age; currently taking medications known to alter taste or smell; having difficulty tasting and smelling; not being in good general health; having a history of choking or difficulty swallowing; having any tongue, cheek, or lip piercing; having dental work in the last month; having smoked tobacco or used nicotine-containing products in the last 30 days; not fluent in English; having a history of any condition involving chronic pain, in their mouth or elsewhere; currently using any prescription pain medication; reaction to aspartame or having phenylketonuria. Informed consent was documented electronically on the first screen of the online survey via a yes/no question.

### 2.3. Sample Preparation

We prepared 8 granular-starch-containing beverages to assess the effect of particle size, concentration, and xanthan content on *Chalky* perception; see Table 1. Pea and potato starches were chosen as they have a different range of particle sizes (mean D4,3 of potato is 42 µm; mean D4,3 of pea is 23.6 µm) but a similar circular/oval shape. We used a two-level factorial design with 10% and 20% *w*/*v* concentration of pea (Purity P 1002 Lot: BBF0065, Ingredion (Bridgewater, NJ, USA)) and potato (PENPURE 10L Lot: 2104000102, Ingredion (Bridgewater, NJ, USA)) starches and xanthan gum (Bob’s Red Millis (Milwaukie, OR, USA)) (0.075%, 0.15% *w*/*v*). A minimal amount (Table 1) of aspartame (Spectrum A1377, Lot No. 1LC0739) and vanillin (Sigma-Aldrich W310700 (St. Louis, MO, USA)) was added to the beverages to make them more palatable for participants. The beverages were prepared in reverse-osmosis (RO) water by blending xanthan gum, aspartame, vanillin, and starch in a Vitamix blender Model Pro-750 (Cleveland, OH, USA).

### 2.4. Design and Rationale

Ratings were collected in Compusense20 software (Guelph, ON, Canada) to understand the usage of the term *Chalky* relative to other terms that could be used to describe the perception of fine particles in a model beverage where the granular starch content was systematically varied. Specifically, participants were asked to rate *Chalky*, *Powdery*, *Gritty*, *Sandy*, *Mouthdrying*, and *Residual Mouthcoating* to evaluate differences in their perception of these attributes for the sample beverages. These terms were selected from recent work by our group (e.g., [30]) and others (e.g., [31]), as well as an informal tasting of samples by three of the authors; final attributes were then piloted with ~10 members of our research group to confirm their appropriateness for these specific samples.

Prior to tasting any samples, participants were asked to provide a saliva sample by chewing on a 5 × 5 cm square of parafilm (Parafilm “M”, American National CAL, Chicago, IL, USA) for 5 min while spitting in a pre-weighed tube every 30 s to measure their salivary flow rate [32]. They then completed an orientation to a general labeled magnitude scale (gLMS) [33]. The gLMS is anchored with no sensation and the strongest imaginable sensation of any kind, with additional semantic labels for barely detectable, weak, moderate, strong, and very strong at specific points [34]. To understand changes in perception during and after consumption, participants were asked to complete a multi-point consecutive consumption test that was modified from the previously described sequential profiling method [31]. Here, they were asked to consume each sample beverage in small aliquots (5 mL), labeled with three-digit codes, a total of 3 times, making ratings on a gLMS at 0, 30, and 60 s after each sip, for a total of 9 timepoints per sample. Before tasting any test samples, they were given a warm-up sample, made of a mix of pea and potato starch at 10% *w*/*v* concentration and 0.075% *w*/*v* xanthan gum, to minimize any first-sample effect. They were also instructed to hold the sample in their mouth for 5 s before swallowing the entire sample. The order of presentation of test samples was randomized. To reduce fatigue, only 4 samples were given in a single session and sessions were separated by at least 2 days. Between each sample, there was a forced 2 min break for participants to clean their palate with RO water. At the end of the second session, they were asked to define *Chalky*. Demographics (self-reported age, gender, race, and ethnicity) were collected at the end of the second session, as well as the consumption frequency of protein shakes or supplemental nutrition products.

### 2.5. Dispersion Viscosity Measurement

The dispersion viscosity data of the starch suspensions were recorded at 37 °C using a strain rheometer (ARES 400601.901, TA Instruments, New Castle, DE, USA) with a parallel plate geometry. This temperature was chosen to match the mouth temperature during consumption. A sweep test was conducted with decreasing shear rate from 10 to 0.1 s^−1^ in logarithmic function. The measurements were made in clockwise and counterclockwise directions, lasting about 7 min. All measurements were taken in triplicate.

### 2.6. Data Analysis

Data were analyzed using RStudio (version 2023.03.1). A linear mixed-effect model was applied to the *Chalky* ratings using ‘lme4’ [35]; fixed factors included particle size, concentration, xanthan content, salivary flow group, sip time, and rating timepoint. Participants were split into two groups (high or low salivary flow) according to the median average salivary flow rate using the measurements of the weights of their saliva on both test days. This was to test if salivary flow rate influenced *Chalky* ratings. Participants were a random factor in the model to take individual differences in the use of scale rating into account. Outliers more than 2.5 standard deviations from the mean residuals were removed to increase the normality of residuals. Interactions up to three-way ineractions were included in the model. A post-hoc comparison of estimated marginal means of significant interactions was made using ‘emmeans’ [36]. The effect size of all the factors in the model was calculated using ‘effectsize’ [37]. A decorrelation analysis [38] comparing *Chalky* ratings to other ratings was carried out by correlating the mean of the perceptions, grouped by sample beverage. Principal Component Analysis (PCA) was performed on the mean sensory ratings of each sample beverage and the PCA loadings plot was made using “SensomineR” [39], “plotrix” [40] and “ggrepel” [41]. Line plots of the estimated marginal means of sensory perception intensity and consumption time points were generated using the “ggplot2” package [42]. A statistically significant difference was defined as *p* < 0.05.

## 3. Results

### 3.1. Participants Characteristics

Participant demographics are summarized in Table 2. Initially, 99 participants registered for the sensory test; however, 10 participants only completed half of the test, so they were removed prior to data analysis. Another seven participants were removed as they failed the gLMS orientation. The criteria for using the gLMS as intended were based onthe proper ordering of the remembered light and sound sensations: the brightest light they had ever seen > brightness of a well-lit room > brightness of a dimly lit room; loudest sound you have ever heard > loudness of a conversation > loudness of a whisper. This allowed for a deviation of up to 5.0 units on a 100-point scale [43]. No formal power calculation was performed a priori; the sample size was based on the field norms and current guidelines for data collected from untrained consumers [44].

**Figure 1:**
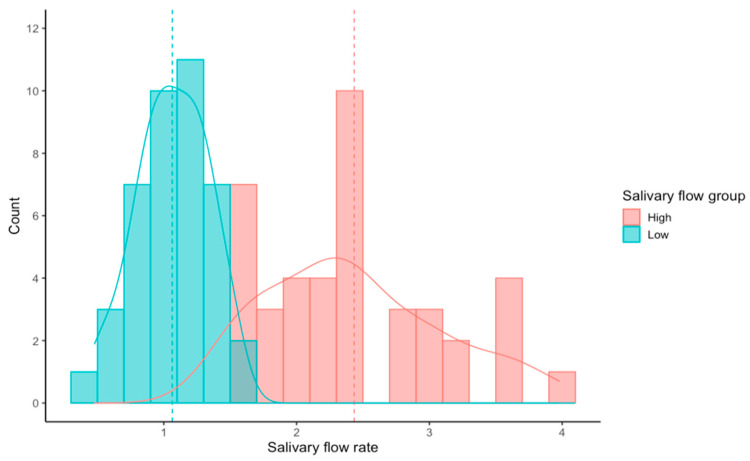
Salivary flow rate of participants in the high- and low-salivary-flow-rate groups. Dotted lines are means of each group.

We saw large individual differences in the salivary flow rate of our participants, varying from 0.48 g/min to 3.98 g/min. The effect of salivary flow rate on Chalky ratings was tested by grouping participants into a high-flow group (mean = 2.43 ± 0.66 g/min) and a low-flow group (mean = 1.06 ± 0.27 g/min). The distribution of flow rates for our participants is shown in Figure 1. The low-flow group had a narrow normal distribution while the high-flow group was more variable, with the distribution skewed to the right.

### 3.2. Effect of Particle Size, Concentration, and Xanthan Content on Chalky Ratings

Particle size [F (1,5626) = 269.4; *p* = <0.001] and concentration [F (1,5626) = 93.9; *p* < 0.001] each had a significant main effect on *Chalky* ratings. Xanthan content did not show evidence of a main effect on *Chalky* ratings [F (1,5626) = 3.5; *p* = 0.0613], but there were significant interactions with xanthan content by size [F (1,5626) = 81.0; *p* < 0.001] and concentration [F (1,5626) = 142.8; *p* < 0.001]. The three-way interaction size by concentration by xanthan content was also significant [F (1,5626) = 43.0; *p* < 0.001].

**Figure 2:**
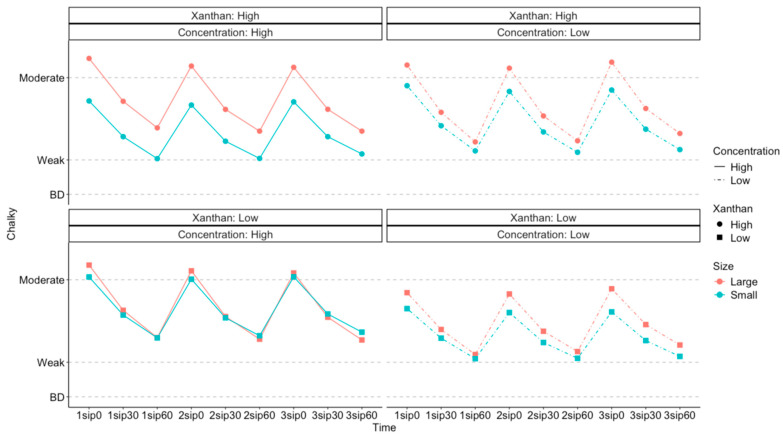
Estimated marginal means of *Chalky* ratings (*y* axis) across all participants, showing how the effect of particle size differed with xanthan content and concentration. The *x* axis shows each sip (1 to 3) and the corresponding time for rating the sensory perceptions (0, 30, and 60 s after each sip).

**Figure 3:**
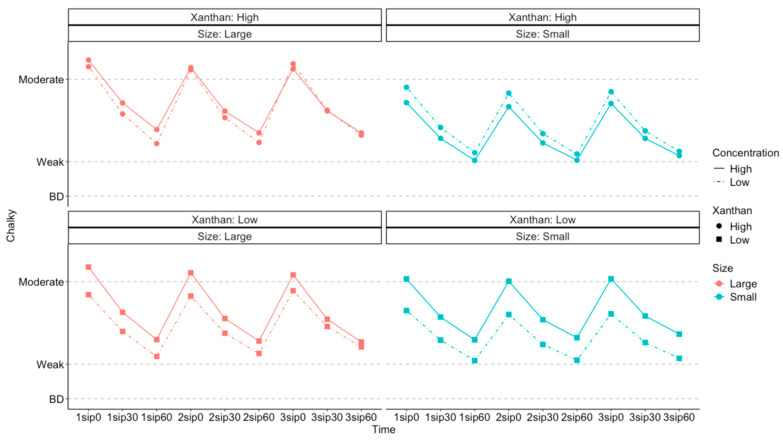
Estimated marginal means of *Chalky* ratings (*y* axis) across all participants, showing how the effect of concentration differed with xanthan content and particle size. The *x* axis shows each sip (1 to 3) and the corresponding time for rating the sensory perceptions (0, 30, and 60 s after each sip).

To facilitate interpretation of the interactions, estimated marginal means of *Chalky* ratings for concentration, particle size, and xanthan content are presented in Figure 2 and Figure 3. The differential effect of particle size (*p* < 0.001) on *Chalky* ratings was greatest in the high xanthan content and at a high concentration level (Figure 2, top left). Conversely, with a low xanthan content but a high concentration (Figure 2, bottom left), there was no evidence (*p* = 0.37) of a difference between the particle sizes. When particle concentration was low (right side of Figure 2), particle size influenced *Chalky* ratings, but xanthan content did not appear to alter this effect. Therefore, we can conclude that a larger particle size tends to lead to a higher *Chalky* rating in general, but this difference is minimized at high concentrations and low xanthan contents.

Regarding concentration effects, the differential effect (*p* < 0.001) on *Chalky* ratings was greater in the low-xanthan-content level (bottom of Figure 3). Conversely, with a high xanthan content, there was less of a difference between the concentration levels (top of Figure 3). With a smaller particle size (right side of Figure 3), the differential effect of particle concentration was greater. Therefore, we can conclude that higher particle concentration level tends to lead to a higher *Chalky* rating, but this difference is larger when xanthan content is low.

### 3.3. Salivary Flow Rate Effect on Chalky Ratings

**Figure 4:**
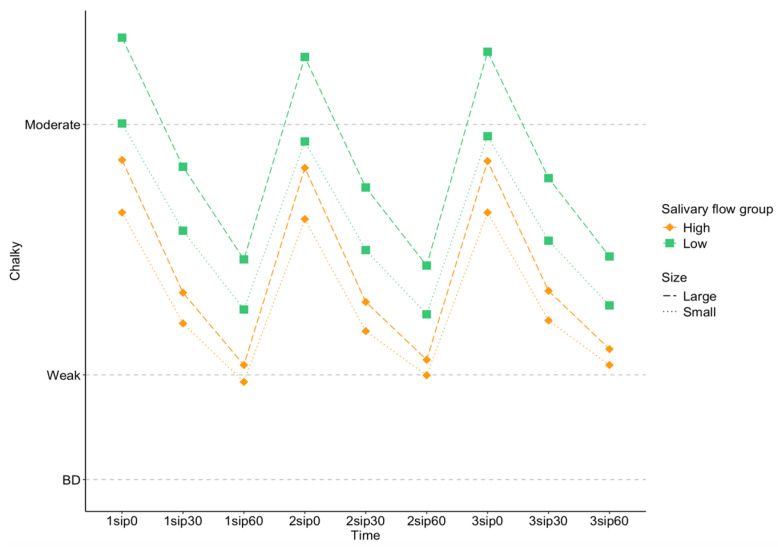
Estimated marginal means of *Chalky* ratings, split into high- (orange diamonds) and low- (green squares) salivary-flow groups as a function of large (dashed lines) and small (dotted lines) particle sizes. The *x* axis shows each sip (1 to 3) and the corresponding time for rating the sensory perceptions (0, 30, and 60 s after each sip).

**Figure 5:**
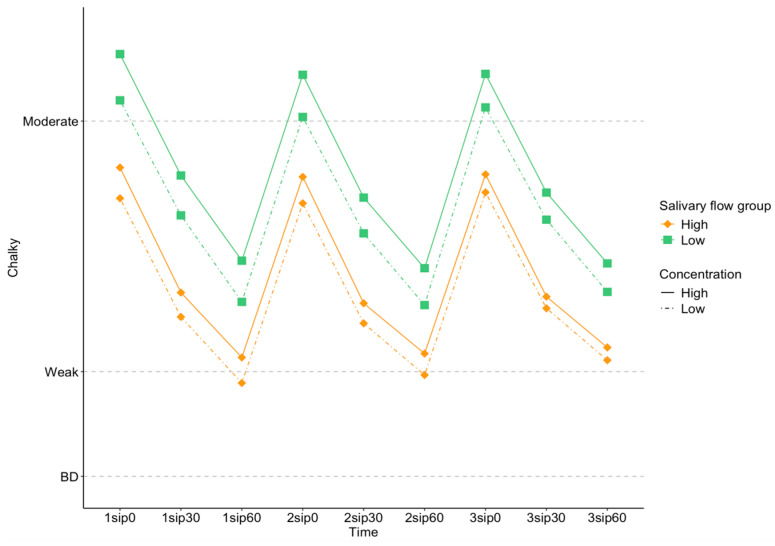
Estimated marginal means of *Chalky* ratings, split into high- (orange diamonds) and low- (green squares) salivary-flow groups as a function of high (solid lines) and low (dashed dotted lines) particle sizes. The *x* axis shows each sip (1 to 3) and the corresponding time for rating the sensory perceptions (0, 30, and 60 s after each sip).

Salivary flow affects texture perception by acting as a lubricant in the mouth. Here, we saw a significant effect of salivary flow on *Chalky* ratings [F (1,80) = 3.97; *p* = 0.0497], with the lower salivary flow rate group reporting higher *Chalky* ratings. There was also significant interaction between salivary flow and particle size [F (1,5626) = 30.6; *p* < 0.001] and salivary flow and concentration [F (1,5616) = 6.72; *p* = 0.0096] on *Chalky* ratings, as shown in Figure 4 and Figure 5.

As shown in Figure 4, larger particles had higher *Chalky* ratings than smaller particles for all participants, but the size of this difference was greater for the low-flow group than the high-flow group.

As shown in Figure 5, a higher concentration lead to greater *Chalky* ratings than lower concentrations for all participants but the size of this difference was greater for the low-flow group than the high-flow group. While differences in *Chalky* between salivary flow rate groups were significant for both Figure 4 and Figure 5, the effect of salivary flow is more pronounced for particle size than for concentration, with the partial Eta^2^ of the interaction between salivary flow and size (0.005) is larger than that of salivary flow and concentration (0.001), consistent with the differences visualized in Figure 4 and Figure 5.

### 3.4. Chalky Ratings over Time

**Figure 6:**
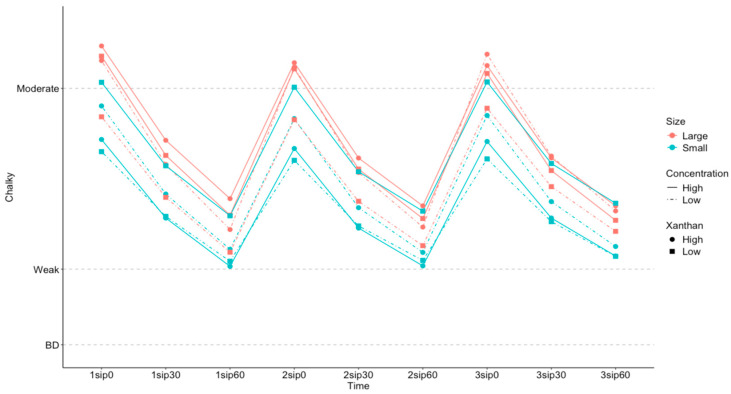
Estimated marginal means of *Chalky* ratings over time for eight samples with different levels of particle size, concentration, and xanthan content across all participants (i.e., collapsed across the high- and low-salivary-flow-rate groups). Labels on the *x* axis are numbered for each sip (one sip to three sips) and the corresponding time of the rating for that sip (0, 30, 60 s after consumption). Both particle concentration and size had substantial effects on *Chalky* ratings.

The *Chalky* ratings were made immediately after each sip (total of three) at 0, 30, and 60 s (total of nine time points). Mean *Chalky* ratings ranged from weak to strong on the gLMS, as shown in Figure 6. In the linear mixed-effect model, the sip order (1, 2, 3) and rating time points (0, 30, 60 s) were added as fixed effects to understand changes in *Chalky* ratings over time. There was significant effect of sip number on *Chalky* ratings [F (2,5626) = 3.84; *p* = 0.0215]; however, this effect was only found with a drop in *Chalky* ratings at Sip 2, not between Sip 1 and Sip 3 (*p* = 0.99), with these having similar estimated marginal means. Additionally, there was no significant interaction effect between sip and timepoints [F (4,5626) = 1.26; *p* = 0.2828]. However, as expected, timepoint had a significant main effect on *Chalky* ratings [F (2,5626) = 1304; *p* < 0.001], as ratings dropped over each 30 s. Collectively, this suggests there was no evidence of a buildup of chalkiness with repeated sips.

### 3.5. Decorrelation Analysis of Chalky and Other Similar Perceptions on Fine Particles

Here, we compare how *Chalky* and other terms were used to describe the samples evaluated by our participants. By correlating the group means of the rating of each of the samples, we find that *Chalky* was strongly correlated with all other attributes, r (6) = +0.83 or above (*p* < 0.01), as shown in Table 3. Specifically, *Chalky*, *Powdery*, and *Residual Mouthcoating* had a high positive correlation of r (6) = +0.99 or above (*p* < 0.01), whereas *Gritty* and *Sandy* had the lowest correlation to *Chalky*, r (6) = +0.90 (*p* < 0.01). Notably, *Gritty* and *Sandy* had a much higher correlation with each other, r (6) = +0.99. The highest correlation with *Mouthdrying* was *Chalky*, r (6) = +0.93 (*p* < 0.01), and *Powdery*, r (6) = +0.91 (*p* < 0.01), with *Gritty* showing the lowest correlation, r (6) = +0.8 (*p* < 0.01)). Collectively, these data suggest three related but distinct sensations: a *Chalky*/*Powdery*/*Residual Mouthcoating* complex, a *Gritty*/*Sandy* complex, and a *Mouthdrying* complex. This interpretation is supported by the PCA biplot shown in the Supplementary Materials.

## 4. Discussion

The mechanism(s) responsible for chalkiness are not well understood, despite its importance as an unfavorable sensation in foods. Other terms, like *Gritty* and *Sandy*, are often used to describe the sensation of fine particles in food, but they are not entirely synonymous with *Chalky*. Here, the usage of *Chalky* and other related terms was compared in a starch beverage model, and we performed a decorrelation analysis [38] to understand how consumers use these terms. *Chalky*, *Powdery*, and *Residual Mouthfeel* were grouped more closely together than *Gritty* and *Sandy*. Notably, *Mouthdrying* was slightly less correlated to *Chalky* in comparison to *Powdery* and *Residual Mouthcoating*, suggesting that *Mouthdrying* is associated with *Chalky* but might not convey the same concept. This is commercially relevant, as *Mouthdrying* is frequently used with *Powdery*/*Chalky* to describe the sensations elicited by food products like whey protein, soy protein, prebiotic beverages, and yogurts [2,3]. Conversely, *Powdery* was highly correlated with *Chalky*, confirming the previous usage of *Chalky* and *Powdery* as interchangeable (or synonymous) terms [17]. Therefore, the similarities in the usage of these terms may imply comparability in the mechanisms that are responsible. Here, we propose that *Chalky* is a sensation that occurs both during consumption and after consumption because it is related to the residual particles left in the mouth. Still, *Chalky* may not strictly imply mouthdrying as any residual particles left in the mouth may also vary in terms of how much drying they induce.

Particle size, concentration, and dispersion viscosity are major factors in the perception of small food particles, so we expected these factors to affect chalky sensation as well. This study revealed that particle size and concentration are major factors influencing the chalkiness of beverages containing granular starch. Additionally, xanthan content, our surrogate for viscosity, influenced the effect size of particle size and concentration on *Chalky*. Our results are comparable with an early study of chalkiness in soy milk [6] in terms of particle concentration, as they found that a higher soy solids concentration increased chalkiness. However, they also reported they were not able to find an effect of viscosity on chalkiness. We speculate that their direct comparison of samples with the same viscosity instead of systematically varying the samples’ viscosity prevented them from identifying the effect of viscosity on chalkiness. Another study looking at the effect of softness and viscosity on gritty/smooth sensations reported that increased viscosity using hydrocolloids increases the smoothness of soft gel particles dispersed in the matrix [10]. When studying the effect of particle size on the tribology of yogurts, however, Laiho et al. [45] could only find a significant increase in grainy sensation with an increase in particle size, but no effect on chalkiness. This may be due to their definition of chalkiness only as an afterfeel (sensation after swallowing), not including sensory perception in the mouth. This suggests that product developers working with insoluble particles could reduce chalkiness by formulating products using a smaller particle size and lower particle concentration.

Our study found a difference in *Chalky* perception between salivary flow groups, as hypothesized. Specifically, those with a lower salivary flow reported higher *Chalky* ratings than the those with a higher salivary flow. We speculate that this occurs due to the more rapid removal of starch particles in individuals with a higher flow. Saliva is known to be a lubricant in the mouth, so the quicker replenishment of saliva presumably helps reduce friction in the mouth in those with a higher salivary flow, although we did not directly measure this here. Elsewhere, the friction coefficient of food such as yogurt and chocolate is reportedly reduced by saliva [28]. Separately, saliva can increase the viscosity of liquid foods, which also can lead to texture perception changes. While many studies have failed to observe associations between salivary flow rate and texture perception [46,47,48], this is likely to be highly food-specific. Our data suggest that salivary flow rate may play a greater role in clearing fine particles in a liquid food relative to the null effects seen for larger particles in semi-solid foods. Practically, this suggests that when developing products for older adults or others experiencing oral dryness, testing needs to be performed in specific foods to avoid overgeneralizing.

Some strengths and limitations should be noted. We were able to capture the changes in *Chalky* ratings between sips and up to 1 min after each consumption, while other studies seldom capture the simulation of the regular consumption of beverages using multiple sips. However, we only included three consecutive sips of the same sample in small aliquots to prevent fatigue, given the large number of samples needed for the factorial design. This choice reduced the number of consecutive sips relative to prior work on the buildup of sensations over time [18,49], precluding direct comparison. Still, this remains a strength, as many past studies on *Chalky* perception only use a single rating. We did not see any buildup of *Chalky* ratings here; we cannot completely rule out whether *Chalky* sensations may still build with longer consumption times, larger sips, and/or an increased number of sips, but our data suggest this is unlikely. Past studies [10,20] have shown the effect of dispersion viscosity on the texture perception of small particles; thus, higher viscosities could be used in aqueous models or semi-solid models in future work on chalkiness.

## 5. Conclusions

This study documents differences in chalky ratings in beverages with different starch granule sizes, concentrations, and xanthan contents. While no build-up of *Chalky* ratings was found over the multi-sip test, the particle size and concentration of the starch each significantly affected *Chalky* ratings, and these interacted with the xanthan content. However, only aqueous beverages were used here as a model, so we cannot speculate about thicker, semi-solid products, like mousse or yogurt. Future studies should use higher-viscosity beverages or semi-solid foods to explore the potential of viscosity to mask these sensations.

We also found evidence of substantial individual differences across participants—those with a low salivary flow rate experienced more *Chalky* sensations than those with a high salivary flow rate. Moreover, the salivary-flow-rate groups showed an interaction with particle size and concentration, suggesting that particle size and concentration affected the chalkiness ratings in the low-salivary-flow-rate group more than the high-salivary-flow-rate group. More work is needed to understand how the low-salivary-flow-rate group perceives small food particles differently. As chalkiness has been found to be associated with low liking in foods [30] and a low salivary flow rate is associated with a stronger perception of chalkiness in beverages, this might imply that low-salivary-flow groups might have a lower liking of chalky beverages. As having a lower salivary flow is prevalent in the elderly, their perception and liking of chalky medical nutritional beverages may be affected, which eventually can lead to malnutrition [50]. Therefore, work is needed to examine the direct impact of salivary flow rate on the liking of chalky beverages, as this might provide insights to improve their acceptability.

## Figures and Tables

**Table 1:** Sample content, including starch type, concentration, xanthan gum, aspartame, vanillin, and dispersion viscosity, for the eight starch beverage samples.

Type of Starch	Starch Concentration (*w*/*v*%)	Xanthan Gum (*w*/*v*%)	Aspartame (*w*/*v*%)	Vanillin (*w*/*v*%)	Dispersion Viscosity (Pa.s at 1/s)
Pea	10	0.075	0.0124	0.1	0.07 ± 0.01
Potato	10	0.075	0.0124	0.1	0.08 ± 0.00
Pea	20	0.075	0.0124	0.1	0.08 ± 0.00
Potato	20	0.075	0.0124	0.1	0.11 ± 0.01
Pea	10	0.15	0.0124	0.1	0.41 ± 0.04
Potato	10	0.15	0.0124	0.1	0.44 ± 0.02
Pea	20	0.15	0.0124	0.1	0.45 ± 0.04
Potato	20	0.15	0.0124	0.1	0.55 ± 0.02

**Table 2:** Participant characteristics include gender, age, and salivary flow rate groups.

Characteristics	Mean ± SD
Gender (32 men, 49 women)	
Age (years, n = 82)	41.7 ± 14.1
Saliva group	
High salivary flow (n = 41, g per min)	2.4 ± 0.7
Low salivary flow (n = 41, g per min)	1.1 ± 0.3

**Table 3:** Correlation matrix (r, df = 6) of sensory perceptions on fine particles using the mean perception intensity of each sample.

	Chalky	Powdery	Sandy	Gritty	Mouthdrying
Powdery	0.99				
Residual Mouthcoating	0.99	1.00	0.93	0.93	0.93
Mouthdrying	0.93	0.91	0.86	0.83	
Gritty	0.90	0.94	0.99		
Sandy	0.90	0.94			

## Data Availability

The original contributions presented in the study are included in the article/Supplementary Material, further inquiries can be directed to the corresponding author.

## Supplementary Materials

The following supporting information can be downloaded at: https://www.mdpi.com/article/10.3390/foods13121852/s1, Figure S1: Principal component analysis (PCA) bi-plot by the covariance matrix of the group mean ratings for the 8 beverage samples.
