# Supplementary material for: What Is Chalky? Investigating Consumer Language and Perception of Fine Particles in Beverages Containing Pea and Potato Starch
Source: Foods. 2024 Jun 13;13(12):1852. doi: 10.3390/foods13121852 (PMC11203002; doi:10.3390/foods13121852)
Supplement: Supplementary file 1 [file foods-13-01852-s001.zip › foods-3029518-supplementary.pdf]

Supplementary materials for Ma et al. *What is Chalky?*

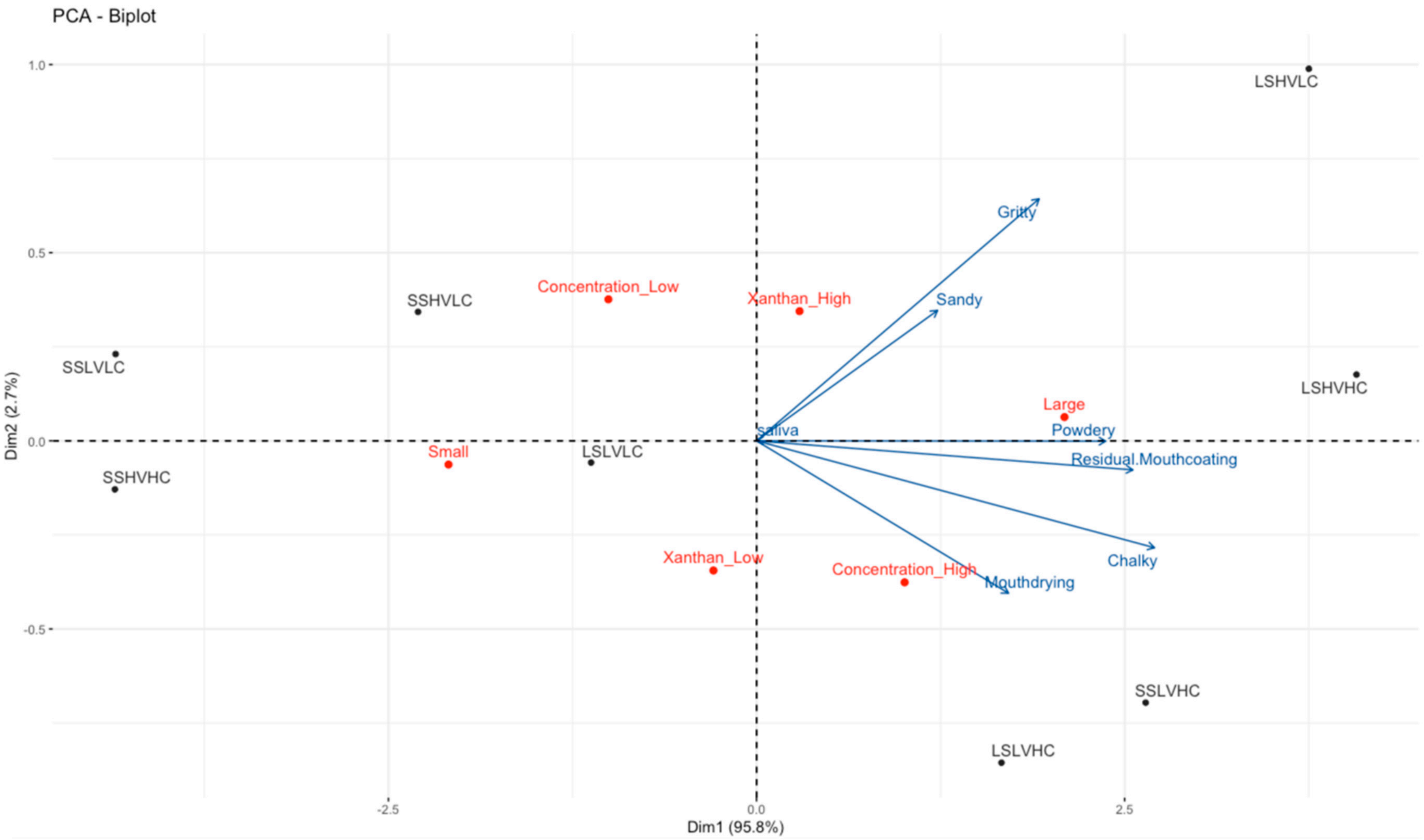

Figure S1. Principal component analysis (PCA) bi-plot of the covariance matrix of the group mean ratings for 8 beverage samples. This figure illustrates the relationship among the samples (black) that were made with different levels of particle size, concentration and xanthan content (red) while the 6 sensory attributes (blue) rated in the study. In the sample names, the first 2 characters are the size, the next 2 characters are the viscosity, and the last 2 characters are concentration. Specifically, LS and SS refer to large and small particle size, with large particles generally falling on the right side of the plot and small particles generally falling on the left. Similarly, HC and LC refer to high and low particle concentration; the low concentration samples tend to fall on the left, with high concentration on the right. Finally, HV and LV refer to high and low xanthan content; the effect of xanthan content is small, but low viscosity samples tend to fall at the bottom with high level of xanthan content at the top. The only notable deviation from this overall pattern is the inversion of the low xanthan content low concentration large particle beverage and the low viscosity high concentration small particle sample. The total explained variance was 98.5%.
